# (*E*)-4-Methyl-2-[(*R*)-1-phenyl­ethyl­imino­meth­yl]phenol

**DOI:** 10.1107/S1600536808013160

**Published:** 2008-05-14

**Authors:** Fang-Fang Dang

**Affiliations:** a School of Science, Xi’an University of Architecture and Technology, Xi’an 710055, People’s Republic of China

## Abstract

In the title Schiff base, C_16_H_17_NO, the dihedral angle between the two aromatic rings is 63.59 (2)°. A strong intra­molecular O—H⋯N hydrogen bond is observed between the hydroxyl group and the imine N atom.

## Related literature

For photochromism and thermochromism of Schiff bases, see: Cohen *et al.* (1964 ▶).
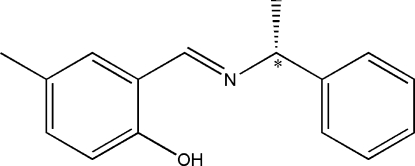


## Experimental

### 

#### Crystal data

C_16_H_17_NO
*M* *_r_* = 239.31
Monoclinic, 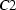

*a* = 20.342 (8) Å
*b* = 5.911 (2) Å
*c* = 14.551 (5) Å
β = 128.585 (4)°
*V* = 1367.7 (9) Å^3^
*Z* = 4
Mo *K*α radiation
μ = 0.07 mm^−1^
*T* = 296 (2) K
0.35 × 0.34 × 0.26 mm

#### Data collection

Bruker APEXII area-detector diffractometer
Absorption correction: multi-scan (*SADABS*; Sheldrick, 1997 ▶) *T*_min_ = 0.971, *T*_max_ = 0.986
5952 measured reflections
1726 independent reflections
1609 reflections with *I* > 2σ(*I*)
*R*_int_ = 0.034

#### Refinement

*R*[*F* ^2^ > 2σ(*F* ^2^)] = 0.033
*wR*(*F* ^2^) = 0.100
*S* = 1.08
1726 reflections
166 parameters
1 restraint
H-atom parameters constrained
Δρ_max_ = 0.16 e Å^−3^
Δρ_min_ = −0.12 e Å^−3^

### 

Data collection: *APEX2* (Bruker, 2004 ▶); cell refinement: *SAINT* (Bruker, 2004 ▶); data reduction: *SAINT*; program(s) used to solve structure: *SHELXS97* (Sheldrick, 2008 ▶); program(s) used to refine structure: *SHELXL97* (Sheldrick, 2008 ▶); molecular graphics: *SHELXTL* (Sheldrick, 2008 ▶); software used to prepare material for publication: *SHELXL97*.

## Supplementary Material

Crystal structure: contains datablocks global, I. DOI: 10.1107/S1600536808013160/ci2590sup1.cif

Structure factors: contains datablocks I. DOI: 10.1107/S1600536808013160/ci2590Isup2.hkl

Additional supplementary materials:  crystallographic information; 3D view; checkCIF report

## Figures and Tables

**Table 1:** Hydrogen-bond geometry (Å, °)

*D*—H⋯*A*	*D*—H	H⋯*A*	*D*⋯*A*	*D*—H⋯*A*
O1—H1⋯N1	0.82	1.89	2.613 (2)	147

**Fig. 1.**
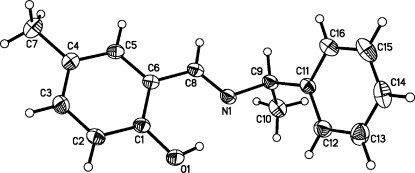
The molecular structure of the title compound, showing 30% probability displacement ellipsoids and the atom-numbering scheme.

## supplementary crystallographic information

### Comment

Compounds presenting photochromism, a reversible colour change brought about in at least one direction, by the action of electromagnetic radiation, attract considerable attention from various fields of chemistry, physics and materials science as potential candidates for practical applications. For a long time, the Schiff bases of salicylaldehyde with aromatic amines (anils or N-salicylideneaniline derivatives) are recognized as such compounds, which undergo enol-keto tautomerism and present common features in their structures and reaction mechanisms. The Schiff base compounds show photochromism and thermochromism in the solid state by proton transfer from the hydroxyl O atom to the imine N atom (Cohen *et al.*, 1964). The tautomerism involves proton transfer from the hydroxylic oxygen to the imino nitrogen atom that occurs intramolecularly *via* a six-membered ring, with the keto species showing bathochromically shifted spectra. Continuing our studies on the relation between the Schiff base geometry in the crystalline state and photochromism and/or thermochromism, we report presently on the crystal structure of the title compound.

The structure of the title molecule is illustrated in Fig. 1. It is a typical salicylaldehyde Schiff base with normal geometric parameters. The C8?N1 bond show the expected double-bond character. The molecule is not planar. The dihedral angle between the two aromatic rings is 63.59 (2)°. A strong intramolecular O—H···N hydrogen bond is observed between the hydroxyl group and the imine N atom (Table 1).

### Experimental

(*R*)-1-Phenylethanamine (0.02 mol, 2.42 g) and 2-hydroxy-5-methylbenzaldehyde (0.02 mol, 2.72 g) were dissolved in ethanol and the solution was refluxed for 3 h. After evaporation, a crude product was obtained which was recrystallized twice from ethanol to give a pure yellow product (yield 82.5%). Calculated for C_16_H_17_NO: C 80.30, H 7.16, N 5.85%; found: C 80.18, H 7.42, N 5.54%.

### Refinement

H atoms were placed in geometrically idealized positions (C—H = 0.93–0.98%A and O—H = 0.82 Å) and constrained to ride on their parent atoms, with *U*_iso_(H) = 1.2*U*_eq_(C) or 1.5*U*_eq_(C_methyl_, O). In the absence of significant anomalous scattering, Friedel pairs were merged prior to the final refinement.

### Crystal data

**Table:**

C_16_H_17_NO	*F*_000_ = 512
*M**_r_* = 239.31	*D*_x_ = 1.162 Mg m^−^^3^
Monoclinic, *C*2	Mo *K*α radiation λ = 0.71073 Å
Hall symbol: C 2y	Cell parameters from 1435 reflections
*a* = 20.342 (8) Å	θ = 1.0–27.6º
*b* = 5.911 (2) Å	µ = 0.07 mm^−^^1^
*c* = 14.551 (5) Å	*T* = 296 (2) K
β = 128.585 (4)º	Block, yellow
*V* = 1367.7 (9) Å^3^	0.35 × 0.34 × 0.26 mm
*Z* = 4	

### Data collection

**Table:**

Bruker APEXII area-detector diffractometer	1726 independent reflections
Radiation source: fine-focus sealed tube	1609 reflections with *I* > 2σ(*I*)
Monochromator: graphite	*R*_int_ = 0.034
*T* = 296(2) K	θ_max_ = 27.6º
φ and ω scans	θ_min_ = 1.8º
Absorption correction: multi-scan(SADABS; Sheldrick, 1997)	*h* = −24→26
*T*_min_ = 0.971, *T*_max_ = 0.986	*k* = −7→7
5952 measured reflections	*l* = −18→18

### Refinement

**Table:**

Refinement on *F*^2^	Secondary atom site location: difference Fourier map
Least-squares matrix: full	Hydrogen site location: inferred from neighbouring sites
*R*[*F*^2^ > 2σ(*F*^2^)] = 0.033	H-atom parameters constrained
*wR*(*F*^2^) = 0.100	*w* = 1/[σ^2^(*F*_o_^2^) + (0.0539*P*)^2^ + 0.1688*P*] where *P* = (*F*_o_^2^ + 2*F*_c_^2^)/3
*S* = 1.08	(Δ/σ)_max_ = 0.001
1726 reflections	Δρ_max_ = 0.16 e Å^−^^3^
166 parameters	Δρ_min_ = −0.12 e Å^−^^3^
1 restraint	Extinction correction: SHELXL97 (Sheldrick, 2008), Fc^*^=kFc[1+0.001xFc^2^λ^3^/sin(2θ)]^-1/4^
Primary atom site location: structure-invariant direct methods	Extinction coefficient: 0.023 (3)

### Special details

**Table:**

Geometry. All e.s.d.'s (except the e.s.d. in the dihedral angle between two l.s. planes) are estimated using the full covariance matrix. The cell e.s.d.'s are taken into account individually in the estimation of e.s.d.'s in distances, angles and torsion angles; correlations between e.s.d.'s in cell parameters are only used when they are defined by crystal symmetry. An approximate (isotropic) treatment of cell e.s.d.'s is used for estimating e.s.d.'s involving l.s. planes.
Refinement. Refinement of *F*^2^ against ALL reflections. The weighted *R*-factor *wR* and goodness of fit *S* are based on *F*^2^, conventional *R*-factors *R* are based on *F*, with *F* set to zero for negative *F*^2^. The threshold expression of *F*^2^ > σ(*F*^2^) is used only for calculating *R*-factors(gt) *etc*. and is not relevant to the choice of reflections for refinement. *R*-factors based on *F*^2^ are statistically about twice as large as those based on *F*, and *R*- factors based on ALL data will be even larger.

### Fractional atomic coordinates and isotropic or equivalent isotropic displacement parameters (Å^2^)

**Table:**

	*x*	*y*	*z*	*U*_iso_*/*U*_eq_	
O1	0.40281 (8)	−0.3392 (2)	−0.02219 (12)	0.0730 (4)	
H1	0.4410	−0.2653	0.0342	0.110*	
N1	0.47315 (9)	0.0034 (2)	0.12256 (11)	0.0538 (3)	
C1	0.32941 (11)	−0.2272 (3)	−0.07580 (15)	0.0532 (4)	
C2	0.25597 (12)	−0.3196 (3)	−0.17537 (17)	0.0641 (5)	
H2	0.2577	−0.4590	−0.2035	0.077*	
C3	0.18064 (12)	−0.2067 (4)	−0.23274 (16)	0.0637 (5)	
H3	0.1323	−0.2725	−0.2990	0.076*	
C4	0.17441 (11)	0.0028 (3)	−0.19481 (15)	0.0573 (4)	
C5	0.24764 (10)	0.0946 (3)	−0.09567 (14)	0.0525 (4)	
H5	0.2451	0.2337	−0.0682	0.063*	
C6	0.32532 (10)	−0.0142 (3)	−0.03530 (13)	0.0475 (3)	
C7	0.09140 (12)	0.1267 (4)	−0.2595 (2)	0.0782 (6)	
H7A	0.1006	0.2860	−0.2597	0.117*	
H7B	0.0674	0.0999	−0.2210	0.117*	
H7C	0.0536	0.0729	−0.3391	0.117*	
C8	0.40074 (10)	0.0940 (3)	0.06644 (13)	0.0509 (4)	
H8	0.3959	0.2336	0.0912	0.061*	
C9	0.54666 (10)	0.1249 (3)	0.22409 (14)	0.0548 (4)	
H9	0.5268	0.2603	0.2390	0.066*	
C10	0.60292 (14)	0.1982 (4)	0.19397 (19)	0.0727 (5)	
H10A	0.5740	0.3084	0.1320	0.109*	
H10B	0.6166	0.0692	0.1687	0.109*	
H10C	0.6537	0.2633	0.2622	0.109*	
C11	0.58971 (10)	−0.0289 (3)	0.33046 (13)	0.0519 (4)	
C12	0.62976 (13)	−0.2262 (4)	0.33758 (17)	0.0664 (5)	
H12	0.6319	−0.2623	0.2772	0.080*	
C13	0.66655 (14)	−0.3697 (4)	0.4330 (2)	0.0802 (6)	
H13	0.6936	−0.5002	0.4366	0.096*	
C14	0.66345 (14)	−0.3215 (5)	0.52172 (19)	0.0864 (7)	
H14	0.6876	−0.4193	0.5853	0.104*	
C15	0.62443 (17)	−0.1278 (6)	0.51633 (19)	0.0894 (8)	
H15	0.6226	−0.0939	0.5771	0.107*	
C16	0.58746 (13)	0.0192 (4)	0.42139 (16)	0.0696 (5)	
H16	0.5612	0.1502	0.4191	0.083*	

### Atomic displacement parameters (Å^2^)

**Table:**

	*U*^11^	*U*^22^	*U*^33^	*U*^12^	*U*^13^	*U*^23^
O1	0.0677 (7)	0.0529 (7)	0.0784 (9)	0.0080 (6)	0.0357 (7)	−0.0145 (7)
N1	0.0630 (8)	0.0471 (7)	0.0489 (6)	0.0001 (6)	0.0337 (6)	−0.0041 (6)
C1	0.0620 (9)	0.0425 (8)	0.0575 (8)	0.0013 (7)	0.0385 (7)	−0.0019 (7)
C2	0.0730 (10)	0.0470 (9)	0.0696 (10)	−0.0048 (9)	0.0432 (9)	−0.0123 (9)
C3	0.0608 (9)	0.0626 (11)	0.0617 (10)	−0.0105 (9)	0.0352 (8)	−0.0081 (9)
C4	0.0601 (9)	0.0568 (9)	0.0627 (9)	0.0030 (8)	0.0421 (8)	0.0094 (8)
C5	0.0650 (9)	0.0448 (8)	0.0604 (9)	0.0031 (7)	0.0453 (8)	0.0026 (7)
C6	0.0610 (8)	0.0407 (7)	0.0508 (7)	0.0016 (6)	0.0398 (7)	0.0022 (6)
C7	0.0649 (10)	0.0782 (15)	0.0904 (13)	0.0100 (11)	0.0479 (10)	0.0140 (12)
C8	0.0671 (9)	0.0418 (7)	0.0516 (8)	0.0007 (7)	0.0409 (7)	−0.0026 (7)
C9	0.0647 (9)	0.0460 (8)	0.0528 (8)	−0.0027 (7)	0.0362 (7)	−0.0084 (7)
C10	0.0849 (12)	0.0704 (12)	0.0686 (11)	−0.0137 (11)	0.0507 (10)	−0.0019 (10)
C11	0.0539 (8)	0.0520 (9)	0.0493 (7)	−0.0096 (7)	0.0320 (6)	−0.0094 (7)
C12	0.0769 (11)	0.0587 (10)	0.0629 (10)	0.0032 (9)	0.0433 (9)	−0.0030 (9)
C13	0.0765 (12)	0.0642 (12)	0.0738 (12)	0.0015 (11)	0.0341 (10)	0.0061 (11)
C14	0.0861 (14)	0.0835 (16)	0.0554 (10)	−0.0224 (13)	0.0273 (10)	0.0057 (11)
C15	0.1092 (17)	0.1044 (19)	0.0575 (11)	−0.0279 (16)	0.0535 (12)	−0.0134 (13)
C16	0.0833 (12)	0.0720 (12)	0.0616 (9)	−0.0097 (10)	0.0492 (9)	−0.0132 (10)

### Geometric parameters (Å, °)

**Table:**

O1—C1	1.350 (2)	C8—H8	0.93
O1—H1	0.82	C9—C11	1.516 (2)
N1—C8	1.274 (2)	C9—C10	1.522 (3)
N1—C9	1.476 (2)	C9—H9	0.98
C1—C2	1.389 (2)	C10—H10A	0.96
C1—C6	1.414 (2)	C10—H10B	0.96
C2—C3	1.377 (3)	C10—H10C	0.96
C2—H2	0.93	C11—C16	1.382 (2)
C3—C4	1.394 (3)	C11—C12	1.389 (3)
C3—H3	0.93	C12—C13	1.383 (3)
C4—C5	1.383 (2)	C12—H12	0.93
C4—C7	1.514 (3)	C13—C14	1.362 (4)
C5—C6	1.397 (2)	C13—H13	0.93
C5—H5	0.93	C14—C15	1.367 (4)
C6—C8	1.457 (2)	C14—H14	0.93
C7—H7A	0.96	C15—C16	1.390 (4)
C7—H7B	0.96	C15—H15	0.93
C7—H7C	0.96	C16—H16	0.93
			
C1—O1—H1	109.4	N1—C9—C10	108.95 (14)
C8—N1—C9	118.78 (15)	C11—C9—C10	113.93 (15)
O1—C1—C2	119.47 (15)	N1—C9—H9	108.6
O1—C1—C6	121.80 (15)	C11—C9—H9	108.6
C2—C1—C6	118.71 (15)	C10—C9—H9	108.6
C3—C2—C1	120.53 (17)	C9—C10—H10A	109.5
C3—C2—H2	119.7	C9—C10—H10B	109.5
C1—C2—H2	119.7	H10A—C10—H10B	109.5
C2—C3—C4	122.18 (18)	C9—C10—H10C	109.5
C2—C3—H3	118.9	H10A—C10—H10C	109.5
C4—C3—H3	118.9	H10B—C10—H10C	109.5
C5—C4—C3	117.16 (17)	C16—C11—C12	117.99 (18)
C5—C4—C7	121.14 (18)	C16—C11—C9	120.77 (17)
C3—C4—C7	121.69 (18)	C12—C11—C9	121.20 (15)
C4—C5—C6	122.38 (16)	C13—C12—C11	121.01 (18)
C4—C5—H5	118.8	C13—C12—H12	119.5
C6—C5—H5	118.8	C11—C12—H12	119.5
C5—C6—C1	119.04 (15)	C14—C13—C12	120.5 (2)
C5—C6—C8	119.88 (15)	C14—C13—H13	119.8
C1—C6—C8	121.07 (15)	C12—C13—H13	119.8
C4—C7—H7A	109.5	C13—C14—C15	119.3 (2)
C4—C7—H7B	109.5	C13—C14—H14	120.3
H7A—C7—H7B	109.5	C15—C14—H14	120.3
C4—C7—H7C	109.5	C14—C15—C16	121.0 (2)
H7A—C7—H7C	109.5	C14—C15—H15	119.5
H7B—C7—H7C	109.5	C16—C15—H15	119.5
N1—C8—C6	122.04 (15)	C11—C16—C15	120.2 (2)
N1—C8—H8	119.0	C11—C16—H16	119.9
C6—C8—H8	119.0	C15—C16—H16	119.9
N1—C9—C11	108.04 (14)		
			
O1—C1—C2—C3	−178.86 (18)	C1—C6—C8—N1	0.4 (2)
C6—C1—C2—C3	−0.5 (3)	C8—N1—C9—C11	123.36 (15)
C1—C2—C3—C4	0.3 (3)	C8—N1—C9—C10	−112.39 (18)
C2—C3—C4—C5	−0.2 (3)	N1—C9—C11—C16	−110.20 (17)
C2—C3—C4—C7	179.20 (18)	C10—C9—C11—C16	128.59 (19)
C3—C4—C5—C6	0.5 (2)	N1—C9—C11—C12	67.34 (19)
C7—C4—C5—C6	−178.95 (15)	C10—C9—C11—C12	−53.9 (2)
C4—C5—C6—C1	−0.8 (2)	C16—C11—C12—C13	−0.2 (3)
C4—C5—C6—C8	178.08 (14)	C9—C11—C12—C13	−177.84 (18)
O1—C1—C6—C5	179.05 (16)	C11—C12—C13—C14	0.7 (3)
C2—C1—C6—C5	0.8 (2)	C12—C13—C14—C15	−0.8 (3)
O1—C1—C6—C8	0.2 (2)	C13—C14—C15—C16	0.5 (4)
C2—C1—C6—C8	−178.06 (16)	C12—C11—C16—C15	−0.1 (3)
C9—N1—C8—C6	179.41 (13)	C9—C11—C16—C15	177.52 (19)
C5—C6—C8—N1	−178.41 (14)	C14—C15—C16—C11	0.0 (3)

### Hydrogen-bond geometry (Å, °)

**Table:**

*D*—H···*A*	*D*—H	H···*A*	*D*···*A*	*D*—H···*A*
O1—H1···N1	0.82	1.89	2.613 (2)	147
